# Sex-based differences in central and cerebral hemodynamic responses to lower body negative pressure (LBNP): an experimental study

**DOI:** 10.1007/s00421-026-06313-7

**Published:** 2026-06-29

**Authors:** F. Tripoli, L. Ridolfi, M. Romanelli, R. Rabbito, S. Roatta, S. Scarsoglio

**Affiliations:** 1 Department of Mechanical and Aerospace Engineering, Politecnico Di Torino, Corso Duca Degli Abruzzi 24, 10129 Turin, Italy; 2 Department of Environmental, Land and Infrastructure Engineering, Politecnico Di Torino, Corso Duca Degli Abruzzi 24, 10129 Turin, Italy; 3 PolitoBioMed Lab, Politecnico Di Torino, Corso Duca Degli Abruzzi 24, 10129 Turin, Italy; 4 Laboratory of Integrative Physiology, Department of Neuroscience, Università Di Torino, Corso Raffaello 30, 10125 Turin, Italy

**Keywords:** Cardiovascular hemodynamics, Cerebral hemodynamics, Countermeasure, Lower body negative pressure, Head-down, Space medicine

## Abstract

**Purpose:**

The effectiveness of lower body negative pressure (LBNP) as an in-flight countermeasure against cardiovascular deconditioning is still debated, with significant gaps in understanding cerebral hemodynamics and sex-related differences. This study investigates acute central, peripheral, and cerebral responses to graded LBNP during exposure to -6° head-down tilt posture (HDT) in 30 subjects (15 females, 15 males).

**Methods:**

Central and peripheral hemodynamic variables (heart rate, blood pressure, cardiac output, and total peripheral resistance) were continuously and non-invasively measured using finger photo-plethysmography along with NIRS parameters from the forearm and the forehead, and cerebral blood velocities within the middle and posterior cerebral arteries (MCA and PCA). The protocol included a 15-min baseline phase in -6° HDT followed by three 5-min levels of LBNP (from -20 to -40 mmHg).

**Results:**

LBNP primarily affected central and peripheral hemodynamics. At the cerebral level, significant changes in cerebral blood velocity and Beer-Lambert NIRS parameters were detected, while spatially resolved spectroscopy NIRS variables did not show significant changes. Sex differences were observed in blood pressure, total peripheral resistance, and forearm NIRS parameters, whereas at the cerebral level, sex had a significant effect only on the cerebrovascular conductance of the PCA (*CVC*_*PCA*_).

**Conclusion:**

Our main findings highlight: (i) comparable anterior and posterior cerebral responses, as both blood velocities in the MCA and PCA exhibited a similar decreasing trend at higher LBNP values; and (ii) sex differences occurring primarily in systemic and peripheral cardiovascular responses.

**Supplementary Information:**

The online version contains supplementary material available at https://doi.org/10.1007/s00421-026-06313-7.

## Introduction

Human spaceflight threatens the health and safety of astronauts due to high levels of cosmic radiation and altered gravity. Specifically, weightlessness induces a number of physiological adaptations affecting several systems of the human body, including the cardiovascular system (Buckey 2006; Blaber et al. 2010; Clément 2011; Demontis et al. 2017).

In microgravity, the complete removal of any hydrostatic gradient leads to a redistribution of fluids from the lower body to the thoracic and cranial districts (Buckey et al. 1996; Videbaek and Norsk 1997; Hargens and Richardson 2009; Norsk 2020). As a result, upon exposure to microgravity, increases in venous return, stroke volume (SV), and cardiac output (CO) have been observed (Norsk 2020). Moreover, the headward fluid shift and the concomitant thoracic congestion activate several regulatory mechanisms responsible for a plasma volume reduction (Alfrey et al. 1996), which, in the longer term, may result in cardiac remodeling and atrophy (Levine et al. 1997; Dorfman et al. 2007; Hargens & Richardson 2009; Hughson et al. 2018). Additionally, findings of an altered autonomic function in terms of baroreceptor and cardiopulmonary reflexes have been reported (Eckberg et al. 2010; Mandsager et al. 2015; Norsk 2020). All these adaptations result in a rather complex picture of *cardiovascular deconditioning*, which, even if it is not life-threatening per se, significantly reduces both orthostatic tolerance and exercise capacity.

Given the above considerations, developing effective in-flight countermeasure protocols is crucial to ensure the health and safety of astronauts in preparation for upcoming space missions to the Moon and Mars. In this context, lower body negative pressure (LBNP) is among the most extensively studied countermeasures to date (Hargens & Richardson 2009; Hargens et al. 2013; Harris et al. 2020; Paladugu et al. 2024). The principle of LBNP is to elicit a fluid shift from the upper to the lower body by creating a partial vacuum around the lower extremities, thereby mimicking the gravity-induced fluid shift in the caudal direction upon standing. The degree of venous pooling depends on both the magnitude and the duration of depressurization and results in reduced venous return and central hypovolemia (Goswami et al. 2019).

Although LBNP studies have provided valuable insights into cardiovascular physiology, significant gaps remain in the context of spaceflight applications, with reported data in the literature being inconsistent and contradictory. There are three key points that are unclear.

Firstly, most of the LBNP studies have been conducted in supine posture (Ogoh et al. 2015; Lewis et al. 2015; Kay and Rickards 2016; Kaur et al. 2018; Goswami et al. 2019; Thrall et al. 2021), and only a few studies have considered LBNP and head-down tilt (HDT) posture simultaneously (Tymko et al. 2016; Watkins et al. 2017; Petersen et al. 2019; Whittle et al. 2025). The HDT posture is considered a ground-based analog of microgravity (Watenpaugh 2016) since it is able to reproduce some of its primary effects, including the gravity-induced headward fluid shift. Therefore, in order to investigate the effectiveness of LBNP as an in-flight countermeasure, it is essential to reproduce conditions that closely resemble the space environment.

Secondly, there are inherent difficulties in investigating cerebral circulation, particularly in acquiring deep cerebral circulation measures. This makes the characterization of the cerebral response incomplete. For instance, most studies investigating cerebral circulation associate changes in cerebral blood flow (CBF) with variations in blood velocity in the middle cerebral artery (MCA) (Goswami et al. 2019; Du et al. 2021), thereby neglecting potential heterogeneity in regional CBF during LBNP-induced orthostatic stress. In particular, to date, it is unclear whether there is a difference in the response between the anterior and posterior cerebral circulations, either in microgravity – where measures on the posterior cerebral circulation are absent (Du et al. 2021) – or during orthostatic stress, where conflicting findings are reported in the literature (Deegan et al. 2010; Sato et al. 2012a; Ogoh et al. 2015; Lewis et al. 2015; Kay & Rickards 2016; Tymko et al. 2016; Kaur et al. 2018; Thrall et al. 2021). Furthermore, it is worth noting that among these works, only Tymko et al. (2016) assessed anterior and posterior responses during combined LBNP and HDT. However, their analysis was limited to a single LBNP level (-50 mmHg) and an HDT angle of -45°, whereas angles of -6° and -15° are more commonly used to simulate microgravity.

Finally, due to the high inter-variability among subjects, several parameters could potentially affect LBNP-induced responses and tolerance (Goswami et al. 2019). Among these factors, sex differences represent a key aspect as more females are being recruited for future space missions. Despite many studies showing that females exhibit lower tolerance to the LBNP-induced hypovolemic stimuli (White et al. 1996; Convertino 1998; Fu et al. 2004), the underlying mechanisms are not yet fully clarified. Several hypotheses have been proposed, including reduced vasoconstrictive capacity (Hachiya et al. 2012; Romanelli et al. 2025), differences in adrenergic and autonomic responses (Convertino 1998; Evans et al. 2001; Cheng et al. 2011), and changes in cerebral perfusion regulation (Blaber et al. 2011). However, some studies reported no substantial differences between male and female responses (Kelly et al. 2004; Rosenberg et al. 2021). Being able to quantify sex differences would be of fundamental importance in establishing whether it is necessary to develop different countermeasure protocols between female and male subjects. Furthermore, sex differences are often investigated during progressive hypovolemic LBNP-induced stimuli until the onset of presyncope (Rosenberg et al. 2021; Jarrard et al. 2024), with very few studies considering sex differences during HDT + LBNP stimuli (Patel et al. 2016; Whittle et al. 2025).

To date, only a few studies have simultaneously investigated and combined the three aforementioned aspects: 1) integration of LBNP and HDT; 2) characterization of anterior and posterior cerebral hemodynamics; and 3) sex-related differences. In the context where common presyncope symptoms (which are manifestations of orthostatic intolerance) have been associated with hypoperfusion of the vertebro-basilar system (Sato et al. 2012a), disentangling uncertainties regarding regional CBF responses would be crucial for spaceflight applications, where reduced orthostatic tolerance represents a serious threat to astronauts’ health. Therefore, to effectively implement LBNP as a spaceflight countermeasure, the full integration of the three mentioned aspects is required.

To this aim, in the present study we measured acute central, peripheral, and cerebral responses to different levels of LBNP during short-term microgravity exposure, achieved via -6° HDT posture. Focusing on sex-related differences, we investigated 18 variables. Specifically, together with the most common central and peripheral hemodynamic variables, we analyzed the cerebral response by combining transcranial Doppler (TCD) and near-infrared spectroscopy (NIRS). In particular, both the anterior and posterior cerebral circulation were assessed in order to study the existence of differences in regional CBF regulation. Finally, to account for sex-related differences, the study was conducted on a balanced cohort of 30 young subjects, equally divided between females and males.

## Materials and methods

### Subjects

Thirty volunteers participated in this study, consisting of fifteen females (age: 24.1 ± 2.4 years, weight: 60.1 ± 11.7 kg, height: 166.9 ± 5.7 cm, and BMI: 21.6 ± 4 kg/m^2^) and fifteen males (age: 23.9 ± 2.2 years, weight: 74.9 ± 8.1 kg, height: 177.6 ± 4.0 cm, and BMI: 23.8 ± 2.4 kg/m^2^). Since the participants were recruited in a rather limited period of time, the sample size was based on the availability of the volunteers. All subjects were healthy, with no reported history of cardiovascular disease. All participants were instructed to avoid alcohol and caffeine for 12 h before the experimental session. The study received approval from the Ethics Committee of the University of Torino (no. 0059551, 30 January 2023) in accordance with the Declaration of Helsinki. All subjects were informed of the experimental procedures and provided their written informed consent.

### Measurements

Central and peripheral hemodynamic variables, including arterial blood pressure (ABP), cardiac output (CO), heart rate (HR), and total peripheral resistance (TPR), were continuously and non-invasively measured using finger photo-plethysmography (CNAP System, CNSystems Medizintechnik, Graz, Austria) (Smolle et al. 2015; Flick et al. 2022). These measurements were obtained by placing a finger cuff on the middle finger of the right hand and a calibration cuff at the brachial level of the right arm. A mechanical support was used to maintain the hand at the subject-specific heart level.

Cerebral blood velocities in the M1 segment of the left middle cerebral artery (*V*_*MCA*_) and P1 segment of the right posterior cerebral artery (*V*_*PCA*_) were continuously monitored using TCD ultrasonography (Dolphin/IQ, Viasonix, Ra’anana, Israel). A helmet equipped with 2 MHz motorized probes (Dolphin/XF Robot, Viasonix, Ra’anana, Israel) was employed to detect *V*_*MCA*_ and *V*_*PCA*_ signals. After identifying the left and right middle cerebral arteries with both probes, the position of the right transducer was slightly adjusted towards the inferior and posterior directions, and its depth was increased with respect to its left counterpart to accurately identify *V*_*PCA*_. Additionally, to isolate blood velocity changes from mean arterial pressure (MAP) variations, cerebrovascular conductance indices (*CVC*_*MCA*_ and *CVC*_*PCA*_) were computed by dividing mean *V*_*MCA*_ and *V*_*PCA*_ by MAP, respectively.

Blood volume and oxygenation levels were acquired at both the brain and arm using NIRS (NIRO-200X, Hamamatsu Photonics K.K., Shizuoka, Japan). Specifically, two NIRS probes were placed over the forehead (with 4-cm inter-optode distance) and the right forearm (with 3-cm inter-optode distance). For each probe, changes in concentration of deoxygenated (HHb), oxygenated (O2Hb), and total (THb = HHb + O2Hb) hemoglobin were measured following the standard Beer-Lambert methodology (Delpy et al. 1988). Furthermore, the NIRS device provided spatially resolved spectroscopy (SRS), allowing us to measure the tissue oxygenation index (TOI) and the tissue hemoglobin index (THI), which provide information on cerebral oxygenation and blood volume changes, respectively. These last two parameters are less affected by hemodynamic changes in superficial tissue circulation, making them more specifically related to the deeper (cerebral) tissue, compared to the Beer-Lambert methodology (Canova et al. 2011; Messere and Roatta 2013; Hirasawa et al. 2016; Saglietto et al. 2021; Eleveld et al. 2023). Specifically, the NIRS probes are used with multiple closely spaced detectors. Therefore, by computing the differential signal from the backscattered light collected from the detectors, the SRS NIRS preserves information from deeper tissues and attenuates information from more superficial tissues.

### Experimental protocol

**Fig. 1:**
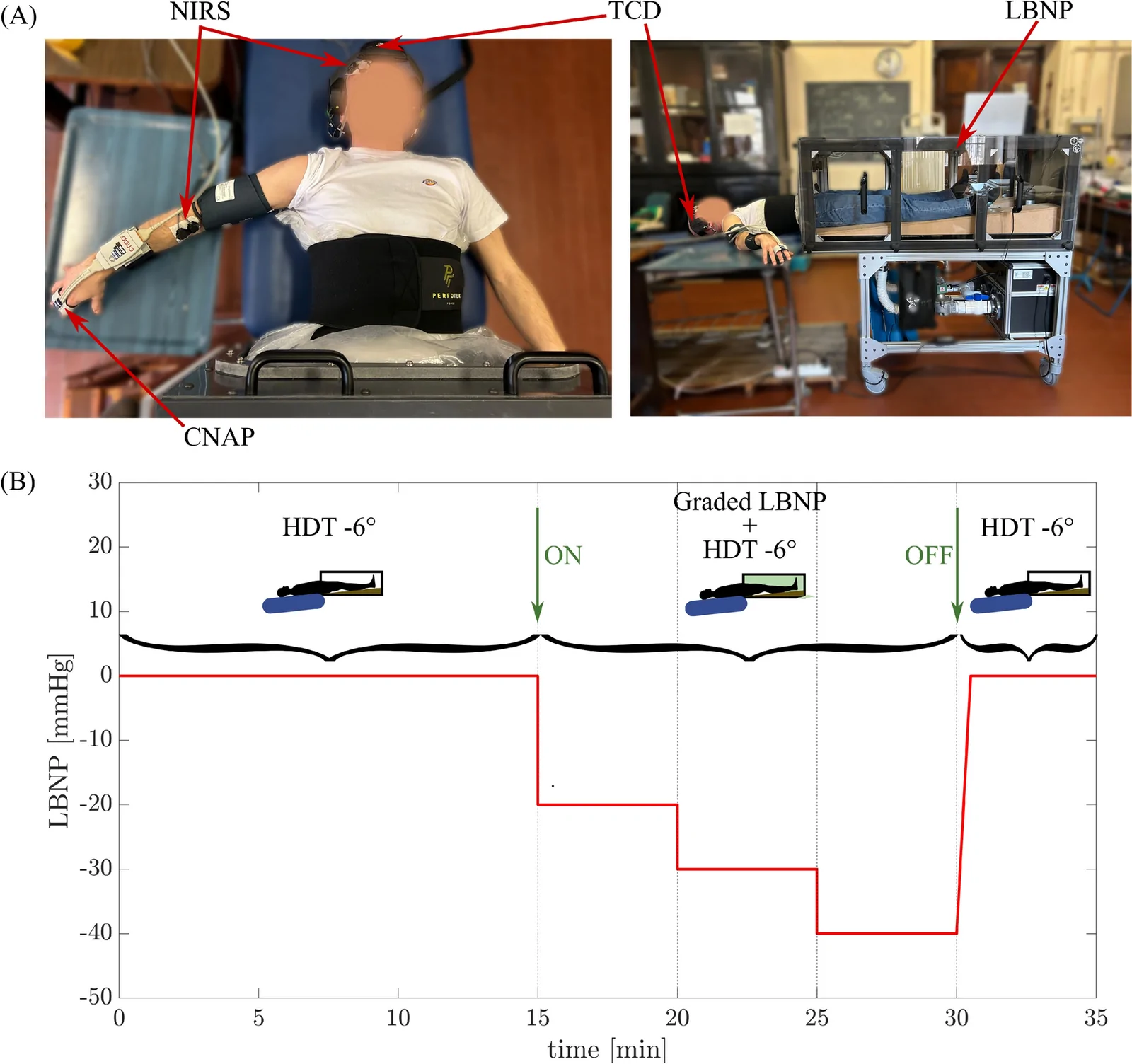
**A** Experimental setup showing the employment of continuous non-invasive arterial pressure monitoring (CNAP), near-infrared spectroscopy (NIRS), transcranial Doppler (TCD), and the chamber for lower-body negative pressure (LBNP); **B** graded LBNP experimental protocol consisting of five different phases (baseline, LBNP at -20 mmHg, LBNP at -30 mmHg, LBNP at -40 mmHg, and recovery). Note the presence of triangular leg supports in the LBNP chamber to maintain the subject in the -6° head-down tilt position for the entire duration of the protocol

Figure 1 shows the setup (Fig. 1A) and the protocol followed during the experiments (Fig. 1B). Before the beginning of the experimental protocol, all subjects remained seated for 15 min to allow familiarization with the experimental setup. The experimental session began with a 15-min baseline phase (BL), during which participants were placed in a supine position at -6° head-down tilt (HDT) within the lower body negative pressure (LBNP) chamber (LBNP 1100, TECHNAVANCE, Austin, TX, USA), which was sealed at the level of the iliac crest. After the 15-min rest period, designed to allow fluid and blood to redistribute, the LBNP session began. This session consisted of a standard graded LBNP protocol with three different levels of negative pressure (-20 mmHg, -30 mmHg, and -40 mmHg), each lasting 5 min. Finally, the protocol included a 5-min recovery session in HDT posture with LBNP turned off. All subjects completed the protocol without experiencing adverse effects. Throughout the five phases, all signals were continuously acquired.

### Data and statistical analyses

Data and statistical analyses were conducted using MATLAB (The MathWorks Inc., MATLAB version R2023b, Natick, MA, USA). All signals were time-averaged over the final two minutes of each experimental phase. To select the optimal window length, a sensitivity analysis was first conducted comparing window lengths of one, two, and three minutes. The two-minute window was selected as it provided the best compromise between detecting the maximum (final) effect and achieving a sufficient noise reduction. Given the interest in analyzing the hemodynamic response with respect to the HDT baseline session – which is considered a terrestrial analog of microgravity (Watenpaugh 2016) – all time-averaged quantities were expressed for each phase as changes (Δ) with respect to their respective time-averaged HDT baseline values. The analysis encompassed two distinct groups: female subjects (F, n = 15) and male subjects (M, n = 15). Data are reported as mean ± SD values.

The Anderson–Darling test was used to verify the normality of the data. Since the null hypothesis of normality was rejected for most of the hemodynamic parameters in at least one of the experimental phases, comparisons between baseline and all other conditions were assessed using the non-parametric Friedman test (Pereira et al. 2015) for each group. Upon reaching statistical significance,* post hoc* analyses were conducted using Wilcoxon signed-rank tests with Bonferroni correction for multiple comparisons (Pereira et al. 2015). In particular, due to the fixed order of the negative pressure steps imposed by our experimental protocol, it was not possible to isolate the effect of pressure from that of time. Consequently, direct comparisons between different LBNP levels (i.e., -20 mmHg vs -30 mmHg, -20 mmHg vs -40 mmHg, and -30 mmHg vs -40 mmHg) are difficult to interpret in terms of pressure alone, so they were not assessed. However, we investigated the hemodynamic responses to graded LBNP-induced hypovolemic stress, which increases progressively with both time and the magnitude of LBNP. To this aim, we expressed each stage of the LBNP graded protocol (i.e., -20 mmHg, -30 mmHg, and -40 mmHg) in terms of the corresponding cumulative stress index (CSI), defined as the cumulative sum of the absolute negative pressures experienced by the subjects multiplied by their corresponding duration (i.e., CSI = $\sum_{i=1}^{n}LBN{P}_{i}\cdot {T}_{i}$; 100 mmHg·min, 250 mmHg·min, and 450 mmHg·min). Then, *post hoc* analyses were limited to comparisons between baseline and each following phase (i.e., BL vs -20 mmHg; BL vs -30 mmHg; BL vs -40 mmHg; BL vs recovery).

Sex differences across the LBNP and recovery phases were assessed for each variable using the Mann–Whitney U-test with Bonferroni correction by considering four comparisons between females and males: at -20 mmHg, -30 mmHg, -40 mmHg, and recovery. A *p*-value of less than 0.05 was considered statistically significant. Additionally, to further explore the effects of orthostatic stress and sex on the systemic and cerebral hemodynamic responses, the following multivariate linear regression model was employed:

$${y}_{i}= {\beta}_{CSI}\left(CSI\right)+{\beta}_{sex}(sex)$$

In this model, CSI was used as a quantitative measure of orthostatic stress, integrating both the intensity and duration of the LBNP stimulus. Sex was included as a categorical variable (F or M). For each hemodynamic parameter, the changes in variables (Δ) were used as dependent variables. Since variable changes were computed with respect to the baseline, only data from the first phase of LBNP (-20 mmHg) to the last phase of LBNP (-40 mmHg) were considered to perform the regression analysis. For the same reason, the model did not include an intercept.

## Results

**Fig. 2:**
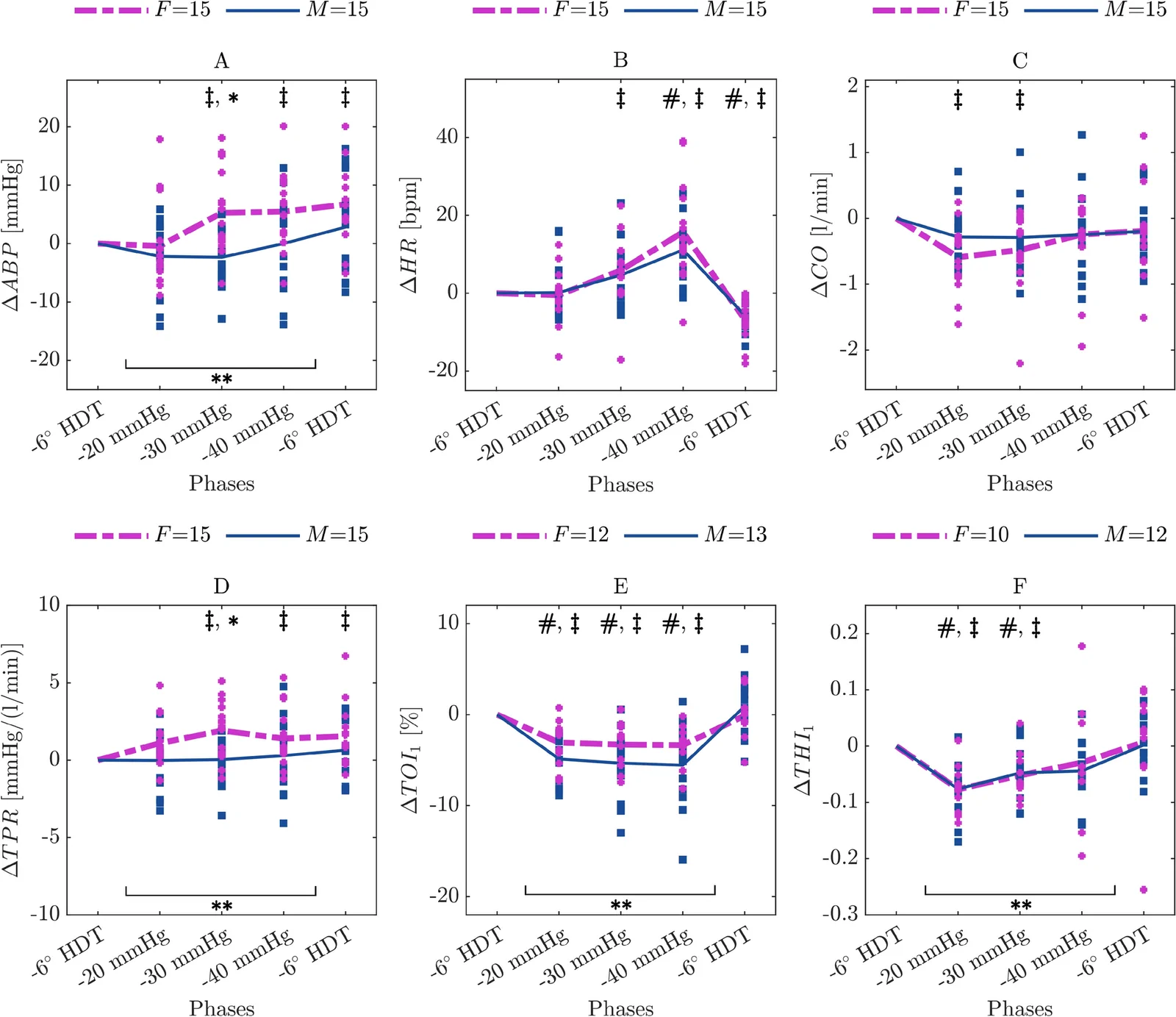
Response of central and peripheral hemodynamic and forearm NIRS variables to graded LBNP. Violet and blue solid curves indicate the mean values obtained during each phase of the protocol for the female and male subjects, respectively. Violet and blue markers refer to the individual time-averaged values of all female and male subjects. Each parameter is expressed as changes (Δ) with respect to HDT baseline values. The number of female and male subjects (F and M) is indicated for each variable. A) Arterial blood pressure (ABP); B) heart rate (HR); C) cardiac output (CO); D) total peripheral resistance (TPR); E) tissue oxygenation index (TOI_1_); F) tissue hemoglobin index (THI_1_). “#” and “‡” denote significant differences with respect to the baseline in the male and female subjects, respectively; “*” and “**” denote significant differences between males and females revealed by the Mann–Whitney U-test and the multivariate linear regression model, respectively

**Fig. 3:**
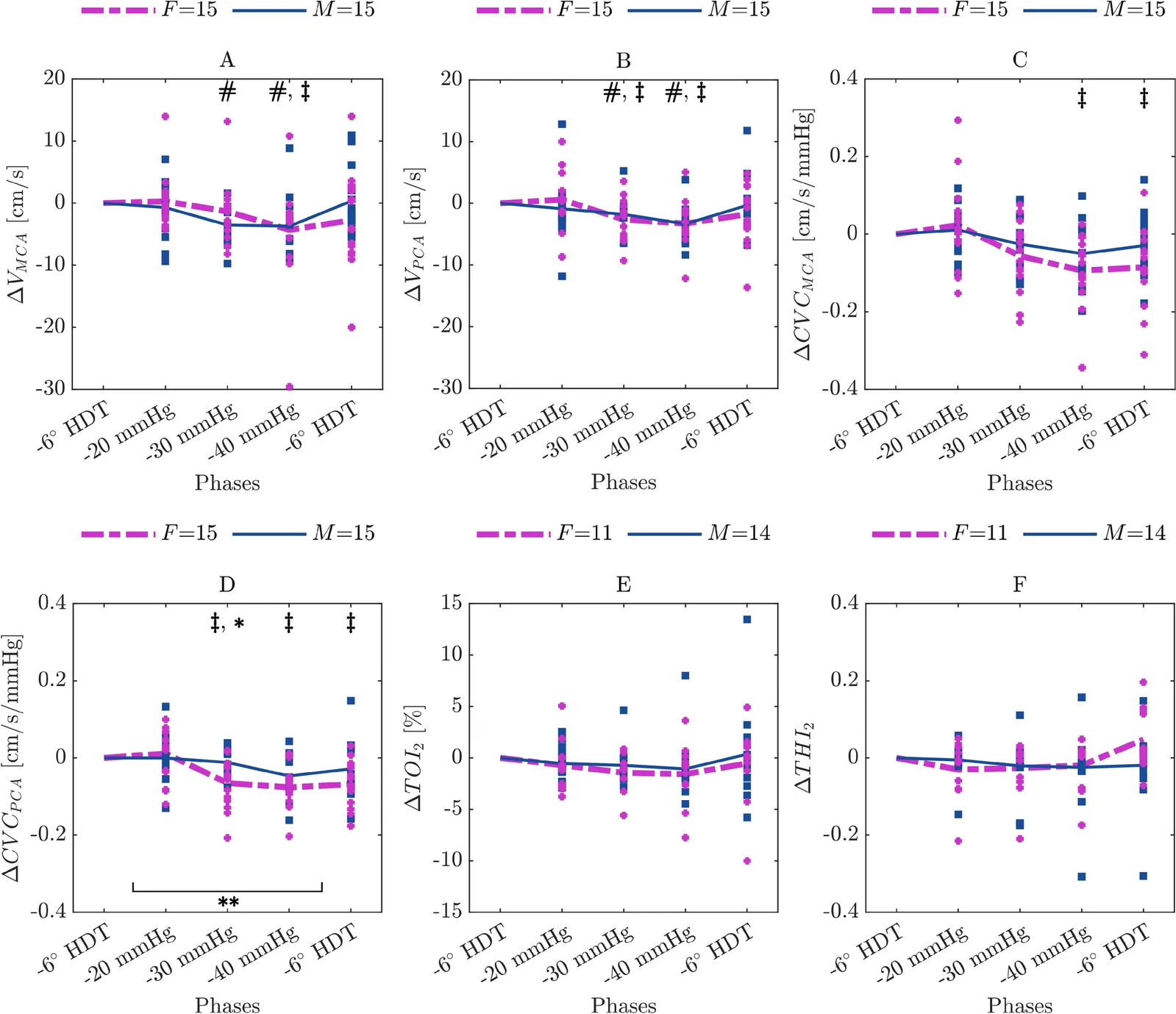
Cerebral variables’ response to graded LBNP. Violet and blue solid curves indicate the mean values obtained during each phase of the protocol for the female and male subjects, respectively. Violet and blue markers refer to the individual time-averaged values of all female and male subjects. Each parameter is expressed as changes (Δ) with respect to HDT baseline values. The number of female and male subjects (F and M) is indicated for each variable. A) Blood velocity in the middle cerebral artery (*V*_*MCA*_); B) blood velocity in the posterior cerebral artery (*V*_*PCA*_); C) Cerebrovascular conductance index in the middle cerebral artery (*CVC*_*MCA*_); D) Cerebrovascular conductance index in the posterior cerebral artery (*CVC*_*PCA*_*)*; E) tissue oxygenation index (TOI_2_); F) tissue hemoglobin index (THI_2_). “#” and “‡” denote significant differences with respect to the baseline in the male and female subjects, respectively; “*” and “**” denote significant differences between males and females revealed by the Mann–Whitney U-test and the multivariate linear regression model, respectively

All participants completed the experimental protocol with no sign of presyncope symptoms. However, due to a malfunction in the NIRS device, all NIRS variables acquired from four subjects (one male and three females) were discarded from the analysis. Additionally, NIRS signals with clear artifacts were excluded (details on the number of subjects included in the analysis for each parameter are reported in Figs. 2 and 3).

In this section, the responses of central and peripheral variables to graded LBNP will first be analyzed. Subsequently, by focusing on both blood velocity and oxygenation parameters, cerebral hemodynamics will be investigated. The time-averaged HDT baseline values, which were used to compute the variable changes (Δ), are reported in Table 1.

**Table 1:** Mean ± SD values of the systemic and cerebral hemodynamic variables measured during the baseline session

Variables	Female	Male
ABP (mmHg)	85.7 ± 14.6	88.3 ± 9.9
HR (bpm)	66.3 ± 11.3	67.2 ± 8.9
CO (l/min)	6.5 ± 1.3	6.7 ± 1.15
TPR (mmHg/l/min)	13.5 ± 3.4	13.9 ± 3.2
TOI_1_ (%)	75.7 ± 3.5	76.7 ± 5.0
*V*_*MCA*_ (cm/s)	80.9 ± 12.8	79.6 ± 8.5
*V*_*PCA*_ (cm/s)	54.7 ± 8.3	53.3 ± 9.1
*CVC*_*MCA*_ (cm/s/mmHg)	0.96 ± 0.22	0.92 ± 0.17
*CVC*_*PCA*_ (cm/s/mmHg)	0.66 ± 0.17	0.62 ± 0.15
TOI_2_ (%)	64.0 ± 9.4	64.9 ± 8.9

The NIRS variables (O2Hb, HHb, THb, and THI) are not included, as they are set to reference values of 0 (for O2Hb, HHb, and THb) or 1 (for THI) at the beginning of each session. Subscripts 1 and 2 denote forearm and forehead measurements, respectively.

### Central and peripheral hemodynamic responses to LBNP in females and males

Figure 2 depicts the response of the central and peripheral hemodynamic variables (ABP, HR, CO, and TPR) to graded LBNP along with the SRS NIRS signal acquired in the forearm (TOI_1_ and THI_1_), whereas Beer-Lambert NIRS parameters (O2Hb_1_, HHb_1_, and THb_1_) together with the raw *p*-values of the Friedman test and *post hoc* comparisons between the different phases of the experimental protocol and the baseline session are reported in Figure SI1 and Table SI1 of the Supplementary Information, respectively. Furthermore, the raw *p*-values of the Mann–Whitney U-test between female and male subjects during the LBNP and recovery phases, and the estimated coefficients and statistics of the multivariate linear regression analysis are presented in Tables SI2 and SI3 of the Supplementary Information, respectively.

In female subjects, the non-parametric Friedman test highlighted statistically significant differences in ABP (Fig. 2A) during the graded LBNP protocol (*p*-value < 0.001), whereas the male subjects did not exhibit statistically significant variations (*p*-value = 0.218). Specifically, *post hoc* analysis showed that females had a significant increase in ABP at -30 mmHg (+ 5.3 ± 7.2 mmHg with respect to the baseline session) and -40 mmHg (+ 6.7 ± 7.0 mmHg with respect to the baseline session). Sex differences were detected by both the Mann–Whitney U-test (at -30 mmHg, see Fig. 2A and Table SI2) and the multivariate regression model (Table SI3). With regard to HR (Fig. 2B), the Friedman test revealed a significant increase in both females and males during LBNP (F: *p*-value < 0.001; M: *p*-value < 0.001). In females, HR increased significantly at -30 mmHg (+ 5.9 ± 8.8 bpm) and -40 mmHg (+ 15.7 ± 12.5 bpm) compared with baseline, whereas in males, significant changes were detected exclusively at -40 mmHg (+ 11.1 ± 8.6 bpm vs baseline). However, neither method used to assess sex differences revealed a significant difference in HR response between females and males. Upon cessation of the LBNP, HR decreased, reaching values that were significantly lower than those of the HDT baseline session in both males and females (M: -5.9 ± 3.5 bpm; F: -7.0 ± 5.1 bpm vs baseline).

Males did not exhibit any significant variation in CO (Fig. 2C) (*p*-value = 0.092) during the graded LBNP protocol. Conversely, in female subjects, the Friedman test indicated a significant effect of LBNP (*p*-value < 0.001), with CO being significantly reduced at -20 mmHg and -30 mmHg. However, at -40 mmHg, no meaningful changes compared to the baseline were observed. Despite these findings, no significant changes were detected between females and males (see Tables SI2 and SI3).

In male subjects, the non-parametric Friedman test revealed no significant changes (*p*-value = 0.74) in TPR (Fig. 2D), while female subjects exhibited meaningful variations (*p*-value = 0.029). In detail, within the female subjects, TPR increased in response to the hypovolemic stimulus elicited by the LBNP application, showing significant differences at -30 and -40 mmHg with respect to the baseline session (as indicated in Table SI1). Furthermore, both the Mann–Whitney U-test (see Table SI2) and the multivariate regression analysis (Table SI3) indicated differences between sexes.

Regarding forearm NIRS variables, the Friedman test detected significant variations in all parameters with the exception of HHb_1_ in female subjects (*p*-value = 0.176; refer to Fig. SI1B and Table SI1 in the Supplementary Information). Notably, O2Hb_1_ (Fig. SI1A) and TOI_1_ (Fig. 2E), both indicators of tissue oxygenation level, exhibited a similar trend in both females and males: after an initial significant decrease at -20 mmHg (M: -127.1 ± 63.3 µM·cm and -4.9 ± 2.6%; F: -84.7 ± 38.9 µM·cm and -3.1 ± 2.5% for O2Hb_1_ and TOI_1_, respectively), both O2Hb_1_ and TOI_1_ remained relatively stable during the subsequent phases of LBNP, whereas an increase in both variables was detected during the recovery session, resulting in values comparable to those initially recorded (refer to *p*-values in Table SI1). Conversely, only in males, HHb_1_ (Fig. SI1B) increased significantly during the exposure to LBNP, with variations at -40 mmHg of + 61.2 ± 71.3 µM·cm. Finally, both indices of blood volume changes, THb_1_ (Fig. SI1A) and THI_1_ (Fig. 2F), showed similar behavior across all phases of the experimental protocol. Specifically, at -20 mmHg, a significant decline was observed in both parameters (M: -100.2 ± 89.2 µM·cm and -0.08 ± 0.05; F: -88.6 ± 44.3 µM·cm and -0.08 ± 0.04 for THb_1_ and THI_1_, respectively). Subsequently, a progressive increase was observed, which was also maintained during the final recovery session, where both parameters were statistically indistinguishable with respect to baseline values (*p*-values = 1.000). Regarding sex differences, the Mann–Whitney U-test (Table SI2) revealed no significant differences between females and males for any NIRS variables. However, the multivariate regression analysis (see Table SI3) indicated that sex had a statistically significant effect on O2Hb_1_, HHb_1_, TOI_1_, and THI_1_.

### Cerebral hemodynamic response to LBNP in females and males

In Fig. 3, the cerebral hemodynamic response to LBNP exposure is depicted by both cerebral blood velocity measures (*V*_*MCA*_ and *V*_*PCA*_), cerebrovascular conductance indices (*CVC*_*MCA*_ and *CVC*_*PCA*_), and SRS NIRS signal acquired in the forehead (TOI_2_ and THI_2_). The response of the Beer-Lambert NIRS parameters (O2Hb_2_, HHb_2_, THb_2_) is shown in Figure SI1 of the Supplementary Information.

Both *V*_*MCA*_ (Fig. 3A) and *V*_*PCA*_ (Fig. 3B) exhibited similar behavior, decreasing significantly during the application of graded LBNP (refer to *p*-values for the nonparametric Friedman’s test in Table SI1). Specifically, the *post hoc* analysis indicated that exposure to -20 mmHg did not affect cerebral blood velocities, with no groups showing statistically significant changes. However, an LBNP level of -30 mmHg resulted in a meaningful reduction in *V*_*MCA*_ compared to baseline values exclusively in males (-3.5 ± 3 cm/s). At -40 mmHg, *V*_*MCA*_ was also significantly reduced in the female subjects (-4.3 ± 8.3 cm/s), whereas this third step of LBNP did not further decrease *V*_*MCA*_ within the male subjects. During the recovery session, *V*_*MCA*_ increased in both groups, returning to baseline values. Contrary to *V*_*MCA*_, the *post hoc* analysis revealed a statistically significant decrease in *V*_*PCA*_ at -30 mmHg in both females and males (M: -1.8 ± 2.6 cm/s; F: -2.7 ± 3.3 cm/s). Exposure to -40 mmHg led to a small further decline in both groups, while the cessation of LBNP restored *V*_*PCA*_ to the initial HDT values. Regarding sex-related differences, no significant effects were detected using the Mann–Whitney U-test or the regression analysis (refer to Tables SI2 and SI3).

In male subjects, graded LBNP had no effect on either cerebrovascular conductance index, *CVC*_*MCA*_ (Fig. 3C) or *CVC*_*PCA*_ (Fig. 3D). In female subjects, *post hoc* analysis revealed a statistically significant reduction compared with baseline for both *CVC*_*MCA*_ (at -40 mmHg and during recovery) and *CVC*_*PCA*_ (at -30 mmHg, -40 mmHg, and during recovery). However, sex differences were observed only for *CVC*_*PCA*_, as shown by both the Mann–Whitney U-test (at -30 mmHg, see Table SI2) and regression analysis (see Table SI3).

In contrast to the forearm, the cerebral NIRS variables exhibited a different behavior between Beer-Lambert (O2Hb_2_, HHb_2_, and THb_2_) and SRS parameters (TOI_2_ and THI_2_), with the former generally being more susceptible to LBNP than TOI_2_ and THI_2_. Specifically, in both females and males, O2Hb_2_ (Fig. SI1D) behaved similarly to O2Hb_1_, showing a significant decrease during the first phase of LBNP (-20 mmHg), after which it remained stable throughout the remaining LBNP phases. Conversely, although the Friedman test indicated a significant effect of the graded LBNP on TOI_2_ (Fig. 3F), the *post hoc* analysis did not reveal any significant differences relative to the baseline session in either group (see Table SI1). During the recovery session, O2Hb_2_ increased in females and males, showing higher meaningful values compared to the baseline, whereas TOI_2_ did not exhibit any significant difference with respect to the initial values. Indices of cerebral blood volume changes, THb_2_ (Fig. SI1F) and THI_2_ (Fig. 3F), provided different results. In particular, THb_2_ mirrored the trend of O2Hb_2_, showing significant changes across all phases and groups, with the exception of the last LBNP step at -40 mmHg in the female subjects. On the contrary, THI_2_ did not present any meaningful changes throughout the entire experimental session, as revealed by the non-parametric Friedman test (M: *p*-value = 0.673; F: *p*-value = 0.468). Notably, neither method used to assess sex differences revealed any significant differences in SRS NIRS variables between females and males (refer to Tables SI2 and SI3), whereas multivariate regression analysis (Table SI3) revealed a statistically significant effect on O2Hb_2_ and THb_2_.

## Discussion

Our experimental study investigated the hemodynamic response to graded LBNP in simulated microgravity conditions, aiming to shed light on the complex cardiovascular adaptations to LBNP-induced hypovolemic stimuli. In particular, our experiments show that: 1) the cerebral hemodynamic response to LBNP is characterized by significant reductions in both MCA and PCA blood velocity, which exhibit a similar behavior with increasing LBNP levels, whereas cerebral SRS NIRS variables (TOI_2_ and THI_2_) do not show significant changes across LBNP levels; and 2) sex differences occur in systemic (ABP) and peripheral hemodynamic responses (TPR, TOI_1_, and THI_1_), and do not appear in the cerebrovascular variables, except for *CVC*_*PCA*_ and NIRS variables related to extracranial circulation (O2Hb_2_ and THb_2_).

### Central and peripheral response

Exposure to LBNP causes a fluid shift toward the lower limbs, resembling the fluid redistribution that occurs during head-up tilt or standing, driven by the gravity-dependent hydrostatic gradient along the body’s longitudinal axis. As a consequence, a series of physiological responses aimed at compensating for the decline in cardiac filling pressure, stroke volume, and cardiac output are triggered (Cooke et al. 2004; Goswami et al. 2019). These responses are primarily governed by both sympathetic and parasympathetic activity, which play a critical role in maintaining adequate arterial blood pressure and tissue perfusion (Cooke et al. 2004; Goswami et al. 2019).

During LBNP, our experiments show an increase in peripheral vascular resistance, as indicated by both TPR and NIRS parameters at the forearm level. However, although the regression analysis (see Supplementary Information) revealed significant CSI effects, sex-specific effects were also detected in these variables and are discussed separately in the dedicated section below. With regard to forearm NIRS parameters, THb_1_ and THI_1_ typically reflect changes in blood volume, whereas TOI_1_ and HHb_1_ provide information about local blood flow and vasoconstriction (Fadel et al. 2004; Fantini et al. 2016; Messere et al. 2017; Canova et al. 2024). Notably, the simultaneous decrease in TOI_1_ (Fig. 2H) and increase in HHb_1_ (Fig. 2F) indicate a reduction in blood flow together with an increase in vascular resistance, which is mainly driven by increased sympathetic activity (Rea and Wallin 1989; Hachiya et al. 2008). These latter changes are dissociated from blood volume changes, which occur at a faster rate, as recently observed in both upper and lower limbs (Romanelli et al. 2025). Furthermore, at the forearm level, the trends observed in SRS and Beer-Lambert parameters are qualitatively comparable, with THI_1_ and TOI_1_ mirroring THb_1_ and O2Hb_1_, respectively. These observations suggest a similar response between superficial and muscle tissues as already observed by Romanelli et al. (2025).

Alongside the increase in sympathetic activity, the significant rise in HR detected during the progression of the LBNP protocol suggests changes in the parasympathetic control (vagal withdrawal). Specifically, at -20 mmHg, HR remains relatively stable with respect to the baseline condition. However, as exposure to LBNP increases from 250 mmHg·min to 450 mmHg·min, HR significantly grows, reaching a variation of about + 24% and + 17% at 450 mmHg·min in females and males, respectively. This rise in HR explains the behavior of CO, which exhibits an initial significant decrease at -20 mmHg that is mitigated at higher LBNP values due to the considerable increase in HR.

### Cerebral response

In the literature, several studies investigated cerebral circulation under LBNP-induced orthostatic stress by measuring the blood velocity in the middle cerebral artery (MCA) through TCD. These studies observed a reduction in CBF despite mean arterial pressure being well above the lower limit of the cerebral autoregulation’s functioning range (Zhang et al. 1998; Lewis et al. 2015; Durocher et al. 2015; Kay and Rickards 2016). Additionally, other experimental studies analyzed CBF in both the anterior and posterior cerebral circulation by simultaneously acquiring blood velocity in the MCA and posterior cerebral artery (PCA), in order to investigate the existence of differences in regional CBF regulation. Notably, the results of these studies show some inconsistencies, with some reporting that the posterior circulation is more preserved than the anterior circulation (Sato et al. 2012a; Ogoh et al. 2015; Kay and Rickards 2016), while others found no differences in regional CBF regulation (Deegan et al. 2010; Lewis et al. 2015; Tymko et al. 2016; Kaur et al. 2018; Thrall et al. 2021). However, all these findings depend on the assumption that the caliber of cerebral arteries does not vary during progressive exposure to LBNP (Purkayastha & Sorond 2013), as usually assumed in TCD studies. However, this hypothesis has been challenged by recent studies based on magnetic resonance imaging, evidencing the occurrence of significant changes in the size of the MCA, such as a 2% reduction during hand grip exercise (Verbree et al. 2017), and a 7% dilatation during hypercapnia (Al-Khazraji et al. 2021), while no significant change was detected during LBNP in an earlier study (Serrador et al. 2000). In this context, in our experimental study, in addition to blood velocity in the MCA and PCA (*V*_*MCA*_ and *V*_*PCA*_, respectively), we measured cerebral oxygenation and blood volume indices via NIRS in order to gain a clearer understanding of the cerebral response during the application of LBNP in HDT posture.

Our results indicate that *V*_*MCA*_ and *V*_*PCA*_ exhibit a similar behavior as the LBNP exposure increases: both decrease progressively during the LBNP protocol, thus suggesting no regional differences under the experimental conditions encountered in our study. This is confirmed by the response of cerebrovascular conductance indices, which show comparable responses in both cerebral arteries across both sexes. Additionally, considering that HDT posture induces a cephalad fluid shift (Ong et al. 2021) – which can alter cerebral circulation in the short-term by transiently increasing CBF compared to the upright posture (Kawai et al. 1993) –, the progressive reductions in *V*_*MCA*_ and *V*_*PCA*_ observed during LBNP (-5% and -6% at 450 mmHg·min in the female and male subjects, respectively, relative to the baseline) suggest that the LBNP-induced hypovolemic stimulus may counteract these HDT-related cerebrovascular changes.

With regard to cerebral NIRS measurements, SRS and Beer-Lambert parameters show different responses. Specifically, while THb_2_ exhibits a strong initial variation following the application of LBNP, similar to THb_1_ at the forearm, no significant changes are detected in THI_2_, suggesting that LBNP does not elicit significant blood volume changes at the cerebral level. These findings corroborate that Beer-Lambert parameters are more affected by changes in the extracranial circulation, which is characterized by a lower autoregulatory capacity than intracranial circulation (Saglietto et al. 2021; Canova et al. 2024). In addition to the different behavior of the two blood volume indices (THI_2_ and THb_2_), our analysis reveals differences also in the oxygenation indices (HHb_2_, O2Hb_2_, and TOI_2_). In particular, although the Friedman test revealed a significant effect of LBNP on TOI_2_,* post hoc* comparisons did not detect any statistically meaningful changes (refer to Table SI1) with respect to the baseline. However, the regression analysis indicated a significant decreasing trend as the LBNP exposure increased. Since, under the hypothesis of constant cerebral metabolism and blood volume, changes in TOI reflect variations in cerebral blood flow (Fantini et al. 2016), the trend observed in TOI_2_ confirms that an actual reduction in CBF took place, as indicated by the reductions in both *V*_*MCA*_ and *V*_*PCA*_. Whether changes in the cross-sectional area of these arteries also took place during LBNP cannot be excluded, based on the present data.

### Sex differences

In the literature, it is commonly recognized that females are less tolerant to LBNP-induced orthostatic stress compared to males (Convertino 1998; Hinojosa-Laborde et al. 2015; Goswami et al. 2019). In general, this lower tolerance in females is associated with reduced stroke volume and cardiac output, along with the onset of pre-syncopal symptoms at lower LBNP intensity than in males (White et al. 1996; Fu et al. 2004; Whittle et al. 2025). Additionally, Romanelli et al. (2025) recently observed a reduced vasoconstrictive response in females compared to males, in terms of reduced slopes of TOI and HHb in both upper and lower limbs, during exposure to mild levels of LBNP, further highlighting sex differences in LBNP-induced cardiovascular response. This difference appears to be maintained in the different conditions of the present study (subjects at -6° HDT rather than in supine position and exposed to a 15-min progressive LBNP rather than to separated 90-s stimuli), as the decrease in TOI_1_ and increase in HHb_1_ tended to be smaller in females than in males. The effects are here attenuated due to the fact that late effects (last 2 min in each pressure step) were considered, rather than the initial slopes (Romanelli et al. 2025). Stronger peripheral vasoconstriction in males has also been reported in other studies (Frey & Hoffler 1988; Hachiya et al. 2012).

This is apparently in contrast with the observed larger increase in ABP and in the derived parameter TPR in females than in males during LBNP. Incidentally, ABP is generally reported to be unaffected by low levels of LBNP and shows a tendency to decrease at higher levels or longer exposure durations, although this effect is attenuated when LBNP is applied in the HDT position (Hall et al. 2024; Whittle et al. 2025). While the significant increase in ABP and TPR in females may be peculiar to the recruited subject group, it is compatible with the above-described limb vasoconstriction if we consider that TPR is also affected by thoraco-abdominal organs. In fact, it has been proposed that females have a more pronounced capacity to constrict these organs as detected by larger changes in bioimpedance measurements during LBNP (Frey & Hoffler 1988; Convertino 1998; Blaber et al. 2013).

Interestingly, in this study, the cerebral response is qualitatively similar between females and males, with no differences observed in both blood velocities and NIRS parameters. However, concerning *CVC*_*PCA*_, significant sex-related differences were revealed both by the Mann–Whitney U-test and regression analysis. Sex differences in cerebrovascular regulation have sometimes been reported, such as in reactivity to CO_2_ (Sato et al. 2012b) and we cannot exclude that LBNP-induced hyperventilation and the ensuing decrease in end-tidal pressure of carbon dioxide (PETCO₂), not measured in this study, might have contributed to the observed decrease in CVC. However, it is unlikely that this has contributed to the sex differences in CVC, considering that no difference was observed in *V*_*MCA*_, *V*_*PCA*_, and TOI_2_ responses. The increased pressor response in females has likely caused a proportionally stronger autoregulatory response in females, resulting in larger decreases in CVC, reaching statistical significance in the occipital circulation.

Overall, these findings suggest that, within the context of our experimental setup, there are no relevant differences in hemodynamic responses to graded LBNP between females and males, with only meaningful variations observed in peripheral hemodynamics and *CVC*_*PCA*_. This may be attributed to the LBNP protocol, which did not include exposure to extreme levels of LBNP (> 50 mmHg). In fact, higher levels of LBNP are known to induce significant orthostatic stress, often leading to the onset of pre-syncopal symptoms and, consequently, promoting sex-specific responses.

### Recovery vs baseline

Following the cessation of LBNP, all subjects underwent a 5-min recovery session while maintaining the HDT posture. During this phase, all variables related to intracranial circulation (TOI_2_, *V*_*MCA*_, and *V*_*PCA*_) and NIRS at the forearm level returned to values that were statistically indistinguishable from baseline. In contrast, the NIRS parameters THb_2_ and O2Hb_2_, during the recovery session, reached values that exceeded those detected during baseline. Considering the absence of change at the cerebral level (as detected by TOI_2_ and THI_2_), this response indicates increased blood volume and, possibly, hyperemia of the extracranial tissues of the face (Canova et al. 2011). This behavior is related to the greater susceptibility to accommodate blood volume changes as well as to weaker autoregulatory mechanisms of the extracranial compared to the cerebral circulation, which is thus more exposed to the transient increase in central venous pressure elicited by increasing blood volume in the thoracic region that occurs as LBNP is turned off. In fact, the cessation of LBNP elicits a similar response to the cessation of the Valsalva maneuver: the increased venous return to the heart results in a further increase in the arterial pressure (Fig. 2A) and a pronounced decrease in HR (Fig. 2B). Notably, this post-LBNP hyperemic phase is absent in the forearm, whereby THb_1_ and O2Hb_1_ return to baseline values without overshoot. This behavior fits with the concept that extracranial tissues of the head are characterized by a weaker sympathetic constrictor tone compared to the upper limbs, as pointed out in recent studies in which no evidence of vasoconstriction was observed in facial tissues during different sympathetic activation tests (Rashid and Roatta 2022, 2024).

### Limitations

A general limitation of this type of study addressing acute exposure to microgravity and LBNP is that observed effects cannot be readily transferred to long-term exposures or treatments. Nevertheless, the use of acute HDT and LBNP allows short-term gravity- and LBNP-induced fluid shifts and long-term chronic adaptations to be discriminated, providing useful insights about adaptation mechanisms and regulatory reflexes.

A further limitation is that adipose tissue thickness (ATT) was not accounted for in the evaluation of NIRS measurements. It has been reported that a larger ATT may attenuate changes in HHb and O2Hb during muscle ischemia (Barstow 2019). In this respect, the smaller changes exhibited by females in response to LBNP could have been caused by a larger ATT, compared to males. Although this possibility cannot be excluded, it is unlikely given that BMI was quite similar in the two groups and, on average, slightly higher in males (23.9 vs. 21.6 kg/m^2^).

An additional limitation may arise from neglecting the effects of the menstrual phase in female subjects, which potentially influences both the autonomic and cardiovascular responses to LBNP-induced hypovolemic stimuli (Shankhwar et al. 2024).

Finally, due to the protocol that was used in this study, care must be taken when comparing the different phases of the LBNP to each other, since the response to a specific level of depressurization is influenced by the previous one.

## Conclusions

The analysis presented in this study addressed three essential aspects that have rarely been investigated simultaneously: integration of LBNP and HDT, characterization of anterior and posterior cerebral hemodynamics, and sex-related differences.

Our findings highlighted no significant differences between anterior and posterior cerebral circulations, as blood velocity in the MCA and PCA exhibited a similar decreasing trend at higher LBNP values.Furthermore, LBNP had a significant effect primarily on variables related to extracranial blood volume and tissue oxygenation changes (O2Hb_2_ and THb_2_), while cerebral SRS NIRS variables did not show significant changes.

Sex-related differences were observed in systemic (ABP) and peripheral (TPR and forearm-derived NIRS measurements) cardiovascular responses, whereas no significant changes were detected at the cerebral level with the exception of *CVC*_*PCA*_. Specifically, a greater reduction in *CVC*_*PCA*_ was observed in females than in males, likely due to an autoregulatory response induced by the significant increase in ABP.

Together, these findings contribute to the understanding of cardiovascular and cerebrovascular responses to graded LBNP during short-term simulated microgravity induced by HDT, providing useful insights into the anterior and posterior cerebral circulation responses and sex-related differences.

## Supplementary Information

Below is the link to the electronic supplementary material.

Supplementary file1 (DOCX 268 KB)

## Data Availability

The raw data are available from the corresponding author upon reasonable request.

## Abbreviations

ABP: Arterial blood pressure
BL: Baseline
CBF: Cerebral blood flow
CSI: Cumulative stress index
CNAP: Continuous non-invasive arterial pressure
CO: Cardiac output
CVC: Cerebrovascular conductance index
HDT: Head-down tilt
HHb: Deoxygenated hemoglobin concentration
HR: Heart rate
ICP: Intracranial pressure
IOP: Intraocular pressure
LBNP: Lower body negative pressure
MAP: Mean arterial pressure
MCA: Middle cerebral artery
O2Hb: Oxygenated hemoglobin concentration
PCA: Posterior cerebral artery
SANS: Spaceflight-Associated Neuro-ocular Syndrome
SRS: Spatially resolved spectroscopy
SV: Stroke volume
TCD: Transcranial Doppler
THb: Total hemoglobin concentration
THI: Tissue hemoglobin index
TOI: Tissue oxygenation index
TPR: Total peripheral resistance
V_MCA_: Blood velocity in the middle cerebral artery
V_PCA_: Blood velocity in the posterior cerebral artery
