# Supplementary material for: Sex-based differences in central and cerebral hemodynamic responses to lower body negative pressure (LBNP): an experimental study
Source: Eur J Appl Physiol. 2026 Jun 29;126(10):5211–24. doi: 10.1007/s00421-026-06313-7 (PMC13633958; doi:10.1007/s00421-026-06313-7)
Supplement: Supplementary file 1 [file 421_2026_6313_MOESM1_ESM.docx]

Supplementary Information

Sex-based differences in central and cerebral hemodynamic responses to lower body negative pressure (LBNP): an experimental study

F. Tripoli,^1^ L. Ridolfi,^2,3^ M. Romanelli,^4^ R. Rabbito,^4^ S. Roatta,^4^ S. Scarsoglio^1,3^

^1^ Department of Mechanical and Aerospace Engineering, Politecnico di Torino, Corso Duca degli Abruzzi 24, Turin, 10129, Italy

^2^ Department of Environmental, Land and Infrastructure Engineering, Politecnico di Torino, Corso Duca degli Abruzzi 24, Turin, 10129, Italy

^3^ PolitoBioMed Lab, Politecnico di Torino, Corso Duca degli Abruzzi 24, Turin, 10129, Italy

^4^ Laboratory of Integrative Physiology, Department of Neuroscience, Università di Torino, Corso Raffaello 30, Turin, 10125, Italy

Correspondence: *Francesco Tripoli (francesco.tripoli@polito.it).*

In Figure SI1, Beer-Lambert NIRS parameters measured at the level of the forearm (O2Hb1, HHb1, and THb1) and forehead (O2Hb2, HHb2, and THb2) are shown. In table SI2 the raw p-values of the Friedman test and post hoc comparisons between the different phases of the experimental protocol and the baseline session are reported. Furthermore, the raw p-values of the Mann-Whitney U-test between female and male subjects during the LBNP and recovery phases, and the estimated coefficients and statistics of the multivariate linear regression analysis are presented in Tables SI2 and SI3, respectively.

**Figure SI1.** Beer-Lambert NIRS parameters’ response to graded LBNP. Violet and blue solid curves indicate the mean values obtained during each phase of the protocol for the female and male subjects, respectively. Violet and blue markers refer to the individual time-averaged values of all female and male subjects. Each parameter is expressed as changes (Δ) with respect to HDT baseline values. The number of female and male subjects (F and M) is indicated for each variable. A) oxygenated hemoglobin concentration in the forearm(O2Hb_1_); B) deoxygenated hemoglobin concentration in the forearm (HHb_1_); C) total hemoglobin concentration in the forearm (THb_1_); D) oxygenated hemoglobin concentration in the forehead (O2Hb_2_); E) deoxygenated hemoglobin concentration in the forehead (HHb_2_); F) total hemoglobin concentration in the forehead (THb_2_); “#” and “‡” denote significant differences with respect to the baseline in the male and female subjects, respectively; “**” denotes significant differences between males and females revealed by the multivariate linear regression model.

**Table SI1.** *p*-values of the Friedman test and post-hoc comparisons between the different phases of the experimental protocol and the baseline session (BL). *p*-values were computed by Wilcoxon signed-rank tests with Bonferroni correction for multiple comparisons. Missing values denote variables that did not pass the non-parametric Friedman test. Bold text indicates *p*-values that are lower than the significance level of 0.05.

| **Variables** | **Male** | | | | | **Female** | | | | |
| --- | --- | --- | --- | --- | --- | --- | --- | --- | --- | --- |
|  | **Friedman** | **BL vs LBNP -20 mmHg** | **BL vs LBNP -30 mmHg** | **BL vs LBNP -40 mmHg** | **BL vs recovery** | **Friedman** | **BL vs LBNP -20 mmHg** | **BL vs LBNP -30 mmHg** | **BL vs LBNP -40 mmHg** | **BL vs recovery** |
| Δ**ABP** | 0.218 | - | - | - | - | **<0.001** | 1.000 | **0.050** | **0.017** | **0.010** |
| Δ**HR** | **<0.001** | 1.000 | 0.255 | **0.001** | **<0.001** | **<0.001** | 1.000 | **0.041** | **0.002** | **<0.001** |
| Δ**CO** | 0.092 | - | - | - | - | **0.001** | **0.001** | **0.013** | 1.000 | 0.675 |
| Δ**TPR** | 0.741 | - | - | - | - | **0.029** | 0.086 | **0.010** | **0.041** | **0.041** |
| Δ**O2Hb_1_** | **<0.001** | **0.002** | **0.004** | 0.064 | 1.000 | **<0.001** | **0.004** | **0.008** | 0.215 | 0.492 |
| Δ**HHb_1_** | **<0.001** | 0.509 | **0.024** | **0.019** | 1.000 | 0.176 | - | - | - | - |
| Δ**THb_1_** | **0.001** | **0.002** | 0.369 | 1.000 | 1.000 | **<0.001** | **0.004** | **0.020** | 1.000 | 1.000 |
| Δ**TOI_1_** | **<0.001** | **0.001** | **0.002** | **0.003** | 1.000 | **<0.001** | **0.006** | **0.004** | **0.002** | 1.000 |
| Δ**THI_1_** | **0.001** | **0.004** | **0.027** | 0.107 | 1.000 | **0.001** | **0.016** | **0.039** | 1.000 | 1.000 |
| Δ***V_MCA_*** | **<0.001** | 1.000 | **0.003** | **0.033** | 1.000 | **0.002** | 1.000 | 0.292 | **0.027** | 0.675 |
| Δ***V_PCA_*** | **<0.001** | 0.606 | **0.021** | **0.006** | 0.750 | **<0.001** | 1.000 | **0.027** | **0.027** | 0.831 |
| *Δ****CVC_MCA_*** | 0.231 | - | **-** | **-** | - | **<0.001** | 1.000 | 0.1414 | **0.001** | **0.027** |
| *Δ****CVC_PCA_*** | 0.158 | - | **-** | **-** | - | **<0.001** | 1.000 | **0.002** | **0.001** | **0.002** |
| Δ**O2Hb_2_** | **<0.001** | **<0.001** | **0.009** | **0.005** | **0.002** | **<0.001** | **0.006** | **0.010** | **0.014** | **0.020** |
| Δ**HHb_2_** | 0.144 | - | - | - | - | 0.055 | - | - | - | - |
| Δ**THb_2_** | **<0.001** | **<0.001** | **0.007** | **0.005** | **0.005** | **<0.001** | **0.006** | **0.020** | 0.137 | **0.020** |
| Δ**TOI_2_** | **0.019** | 0.775 | 0.098 | 0.081 | 1.000 | **0.046** | 0.961 | 0.098 | 0.332 | 1.000 |
| Δ**THI_2_** | 0.673 | - | - | - | - | 0.468 | - | - | - | - |

**Table SI2.** *p*-values of the comparisons between sexes for each phase of the experimental protocol. *p*-values were computed by Mann-Whitney U-tests with Bonferroni correction for multiple comparisons. Bold text indicates *p*-values that are lower than the significance level of 0.05.

| **Variables** | **LBNP -20 mmHg** | **LBNP -30 mmHg** | **LBNP -40 mmHg** | **Recovery** |
| --- | --- | --- | --- | --- |
| Δ**ABP** | 1.000 | **0.017** | 0.390 | 0.931 |
| Δ**HR** | 1.000 | 1.000 | 1.000 | 1.000 |
| Δ**CO** | 0.867 | 1.000 | 1.000 | 1.000 |
| Δ**TPR** | 0.749 | **0.035** | 1.000 | 1.000 |
| Δ**O2Hb_1_** | 0.238 | 0.534 | 0.677 | 1.000 |
| Δ**HHb_1_** | 0.476 | 0.288 | 0.373 | 1.000 |
| Δ**THb_1_** | 1.000 | 1.000 | 1.000 | 1.000 |
| Δ**TOI_1_** | 0.183 | 0.546 | 0.546 | 1.000 |
| Δ**THI_1_** | 1.000 | 1.000 | 1.000 | 1.000 |
| Δ***V_MCA_*** | 1.000 | 0.998 | 1.000 | 1.000 |
| Δ***V_PCA_*** | 0.548 | 1.000 | 1.000 | 1.000 |
| *Δ****CVC_MCA_*** | 1.000 | 1.000 | 0.7491 | 0.5046 |
| *Δ****CVC_PCA_*** | 1.000 | **0.039** | 0.594 | 0.694 |
| Δ**O2Hb_2_** | 1.000 | 1.000 | 1.000 | 0.846 |
| Δ**HHb_2_** | 1.000 | 1.000 | 1.000 | 1.000 |
| Δ**THb_2_** | 1.000 | 0.773 | 1.000 | 1.000 |
| Δ**TOI_2_** | 1.000 | 1.000 | 1.000 | 1.000 |
| Δ**THI_2_** | 1.000 | 1.000 | 1.000 | 0.536 |

**Table SI3.** Regression coefficients (estimate ± SE) and *p*-values for the t-statistic of the independent variables (LBNP levels and sex) in the multivariate regression models for each systemic and cerebral hemodynamic variable. Bold text indicates *p*-values that are lower than the significance level of 0.05.

| **Variables** | **Β_CSI_** | **β_sex_** | ***P*_CSI_** | ***p*_sex_** |
| --- | --- | --- | --- | --- |
| Δ**ABP (mmHg)** | 0.012 ± 0.003 | -4.799 ± 1.286 | **<0.001** | **<0.001** |
| Δ**HR (bpm/min)** | 0.031 ± 0.004 | -2.941 ± 1.694 | **<0.001** | 0.086 |
| Δ**CO (l/min)** | -0.0008 ± 0.0003 | -0.051 ± 0.118 | **0.003** | 0.669 |
| Δ**TPR (mmHg/l/min)** | 0.004 ± 0.0008 | -0.903 ± 0.362 | **<0.001** | **0.014** |
| Δ**O2Hb_1_ (µM·cm)** | -0.108 ± 0.038 | -71.721 ± 15.826 | **0.006** | **<0.001** |
| Δ**HHb_1_ (µM·cm)** | 0.044 ± 0.024 | 35.166 ± 10.002 | 0.076 | **<0.001** |
| Δ**THb_1_ (µM·cm)** | -0.063 ± 0.046 | -34.372 ± 19.212 | 0.173 | 0.078 |
| Δ**TOI_1_ (%)** | -0.008 ± 0.002 | -3.094 ± 0.689 | **<0.001** | **<0.001** |
| Δ**THI_1_ (a.u.)** | -0.00008 ± 0.00004 | -0.035 ± 0.015 | **0.037** | **0.023** |
| Δ***V_MCA_* (cm/s)** | -0.008 ± 0.002 | -0.476 ± 0.986 | **<0.001** | 0.630 |
| Δ***V_PCA_* (cm/s)** | -0.008 ± 0.002 | -0.014 ± 0.708 | **<0.001** | 0.985 |
| *Δ****CVC_MCA_*** | -0.0002 ± 0.00004 | 0.029 ± 0.016 | **<0.001** | 0.077 |
| *Δ****CVC_PCA_*** | -0.0002 ± 0.00003 | 0.026 ± 0.011 | **<0.001** | **0.018** |
| Δ**O2Hb_2_ (µM·cm)** | -0.132 ± 0.031 | -33.671 ± 12.870 | **<0.001** | **0.011** |
| Δ**HHb_2_ (µM·cm)** | 0.029 ± 0.012 | 6.468 ± 4.834 | **0.016** | 0.185 |
| Δ**THb_2_ (µM·cm)** | -0.103 ± 0.030 | -27.203 ± 12.426 | **<0.001** | **0.032** |
| Δ**TOI_2_ (%)** | -0.004 ± 0.001 | 0.170 ± 0.471 | **0.003** | 0.720 |
| Δ**THI_2_ (a.u.)** | -0.00006 ± 0.00004 | 0.0002 ± 0.015 | 0.086 | 0.990 |
